# Effect of Ferrous Ion on Heat-Induced Aroma Deterioration of Green Tea Infusion

**DOI:** 10.3390/molecules26144255

**Published:** 2021-07-13

**Authors:** Ying Gao, Jie-Qiong Wang, Jian-Xin Chen, Fang Wang, Gen-Sheng Chen, Jun-Feng Yin, Yong-Quan Xu

**Affiliations:** 1 Key Laboratory of Tea Biology and Resources Utilization, National Engineering Research Center for Tea Processing, Tea Research Institute Chinese Academy of Agricultural Sciences, Ministry of Agriculture, 9 South Meiling Road, Hangzhou 310008, China; yinggao@tricaas.com (Y.G.); wangjieqiong@tricaas.com (J.-Q.W.); mt.jx.chen@tricaas.com (J.-X.C.); wf@tricaas.com (F.W.); gschen@tricaas.com (G.-S.C.); 2 College of Food Science, Southwest University, Chongqing 400715, China

**Keywords:** green tea infusion, aroma deterioration, ferrous ion, catechins, hydrogen peroxide

## Abstract

Aroma deterioration is one of the biggest problems in processing tea beverages. The aroma of tea infusion deteriorates fast during heat sterilization and the presence of ferrous ion (Fe^2+^) aggravates it. The underlying mechanism remains unveiled. In this study, Fe^2+^ was verified to deteriorate the aroma quality of green tea infusion with heat treatment. Catechins were necessary for Fe^2+^-mediated aroma deterioration. By enhancing the degradation of catechins, Fe^2+^ dramatically increased the production of hydrogen peroxide (H_2_O_2_). Fe^2+^ and H_2_O_2_ together exacerbated the aroma of green tea infusion with heat treatment. GC-MS analysis revealed that the presence of Fe^2+^ enhanced the loss of green/grassy volatiles and promoted the formation of new volatiles with diversified aroma characteristics, resulting in a dull scent of green tea infusion. Our results revealed how Fe^2+^ induced aroma deterioration of green tea infusion with heat treatment and could help guide tea producers in attenuating the aroma deterioration of tea infusion during processing.

## 1. Introduction

**Figure 1:**
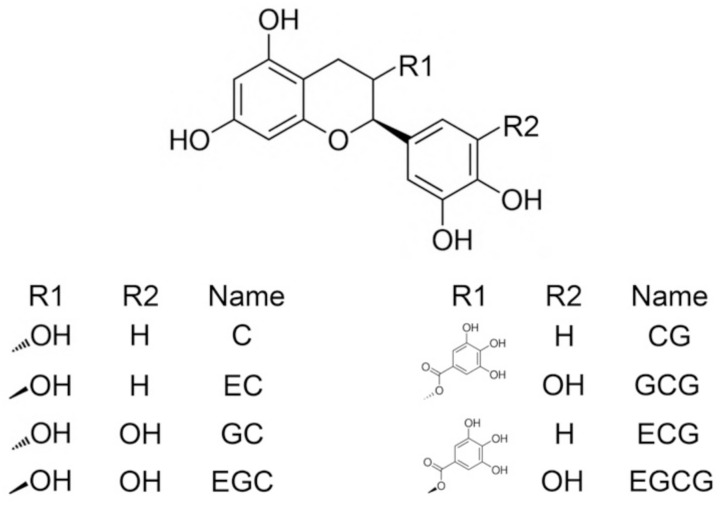
Structural formula of catechins. C, EC, GC, EGC, CG, GCG, ECG, and EGCG are short for (+)-catechin, (−)-epicatechin, (−)-gallocatechin, (−)-epigallocatechin, (−)-catechin gallate, (−)-gallocatechin gallate, (−)-epicatechin gallate, and (−)-epigallocatechin gallate.

Green tea has been traditionally and widely used as a beverage in China. Ready-to-drink tea is preferable in modern society because of its convenience. Compared with freshly-brewed tea, the aroma of ready-to-drink tea is less fresh, sometimes even dull, impairing its sensory quality. The aroma of tea infusion deteriorates fast during heat sterilization [1,2]. Green tea infusion, which contains a higher level of unoxidized catechins (Figure 1), tends to experience more severe aroma deterioration than black tea and dark tea infusions during heat treatment [3]. Aroma deterioration is positively correlated to the degradation of unoxidized catechins and the accumulation of hydrogen peroxide (H_2_O_2_) [4], suggesting catechins might be vital in the aroma deterioration. Nevertheless, there are other factors.

Many metal ions affect the aroma of tea infusion. Among common ions in tea infusion, calcium ion (Ca^2+^), ferrous ion (Fe^2+^), and zinc ion (Zn^2+^) have adverse effects on aroma [5,6]. In particular, Fe^2+^ deteriorates the aroma of tea infusion at concentrations as low as 0.1 ppm [6], implying aroma are very sensitive to Fe^2+^. However, the mechanism of Fe^2+^-mediated aroma deterioration remains unclear. To figure it out, the effects of Fe^2+^ on aroma quality, degradation of catechins, and generation of H_2_O_2_ in green tea infusion with heat treatment were assessed. The roles of catechins and H_2_O_2_ on Fe^2+^-mediated aroma deterioration were explored, respectively. The impacts of Fe^2+^ on volatile profiles were also evaluated. The results would deepen the knowledge of how Fe^2+^ deteriorated the aroma of tea infusion, and provide a new aspect for improving the aroma quality of ready-to-drink tea.

## 2. Results and Discussion

### 2.1. Fe^2+^ Deteriorates the Aroma Quality and Promotes the Degradation of Catechins in Green Tea Infusion with Heat Treatment

The untreated infusion had fresh green tea aroma (defined as “fresh”) (Table 1). Heat treatment of green tea infusion reduced the freshness of the aroma (defined as “slightly dull”). The presence of Fe^2+^ dose-dependently aggravated heat-induced aroma deterioration. It not only enhanced the reduction of the freshness, but also produced an off-aroma of the infusion (defined as “dull”).

Aroma deterioration is associated with degradation of catechins [4]. Catechins are heat-sensitive. When incubated at 100 °C, epimerization was the predominant reaction of epi-form catechins whereas ester hydrolysis was the predominant reaction of GCG [7,8]. In this study, the concentration of total catechins was not significantly changed but the composition of catechins was changed after heat treatment (Table 2).

The concentrations of two epi-form catechins, i.e., EGCG and EC, were decreased after heat treatment. Meanwhile, the concentrations of four non-epi-form catechins (GC, C, GCG, CG) and GA—a degradation product of catechins—were increased. The decrease of EGCG along with the increase of GCG indicated that the epimerization of EGCG took place under this condition. Notably, the loss of EGCG was higher than the increase of GCG. GC was increased though EGC was hardly changed. It suggested that part of GCG further degraded to GC. The change trends of EC and C were similar to that of EGCG and GCG, demonstrating the epimerization of EC also occurred. Taken together, heat treatment promoted the epimerization of EGCG and EC to their corresponding non-epi-forms, and triggered the production of non-gallated catechins. Compared with heated green tea infusion, levels of total catechins, EGCG, GCG, GC, and CG were decreased in heated green tea infusion with Fe^2+^ addition. It implied that Fe^2+^ predominantly promoted the degradation of catechins in green tea infusion with heat treatment.

**Figure 2:**
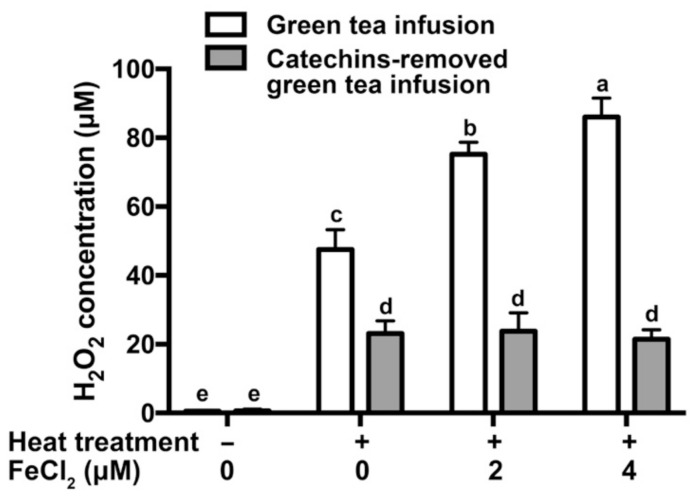
Effect of Fe^2+^ on the accumulation of H_2_O_2_ in green tea infusion with heat treatment. The same letter within each column indicates no significant difference (*p* > 0.05).

The degradation of catechins is coupled to the formation of H_2_O_2_ [9]. In a previous study, the concentrations of catechins in tea infusion were decreased and the concentrations of H_2_O_2_ were increased during storage [4]. A similar phenomenon was detected during heat treatment of tea infusion in this study (Figure 2). The addition of Fe^2+^ dose-dependently increased the levels of H_2_O_2_ in green tea infusion. In contrast, the levels of H_2_O_2_ in catechins-removed green tea infusion were not significantly elevated by the addition of Fe^2+^. It proved that H_2_O_2_ in heated green tea infusion was partially derived from catechins and Fe^2+^ promoted the generation of H_2_O_2_ dependently on catechins.

### 2.2. Effect of Catechins and H_2_O_2_ on Fe^2+^-Mediated Aroma Deterioration in Green Tea Infusion with Heat Treatment

To investigate the roles of catechins on Fe^2+^-mediated aroma deterioration in green tea infusion with heat treatment, the effect of Fe^2+^ on aroma quality of catechins-removed green tea infusion was assessed. According to the results (Table 3), the effect of Fe^2+^ on aroma deterioration was diminished in catechins-removed green tea infusion, implying that catechins were necessary for Fe^2+^-mediated aroma deterioration.

To further test whether catechins participated in Fe^2+^-mediated aroma deterioration via generating H_2_O_2_, the effect of Fe^2+^ on aroma quality of catechins-removed green tea infusion containing exogenous H_2_O_2_ was evaluated. The results (Table 4) revealed that exogenous H_2_O_2_ or Fe^2+^ alone hardly deteriorated the aroma of catechins-removed green tea infusion. However, the combination of exogenous H_2_O_2_ and Fe^2+^ effectively impaired the aroma. It suggested that H_2_O_2_ played an essential role in Fe^2+^-mediated aroma deterioration.

Previous studies have mentioned that Fe^2+^ can react with H_2_O_2_ to generate highly reactive radicals (e.g., hydroxyl radicals) [10]. Fe^2+^ can catalyze the reaction of H_2_O_2_ and superoxide ion to generate hydroxyl radicals and hydroxide ions [10]. Due to the massive production of reactive radicals, the two reactions play important roles in the degradation of various organic compounds, including hydrocarbons, alcohols, and organic acids [11]. Both H_2_O_2_ and superoxide ion are generated during the degradation of catechins [9]. Therefore, it is possible that Fe^2+^ also relies on these two reactions to induce aroma deterioration of green tea infusion. EGCG, the most abundant catechin in green tea infusion, was capable of enhancing the efficiency of Fe^2+^-mediated oxidative systems on the degradation of organic compounds, probably by accelerating the transformation of Fe^3+^ to Fe^2+^ [12]. This implied that catechins might not only participate in Fe^2+^-mediated aroma deterioration by generating H_2_O_2_, but also by promoting the regeneration of Fe^2+^. However, further experiments are needed to verify this hypothesis.

### 2.3. Effect of Fe^2+^ on the Volatile Profile of Green Tea Infusion with Heat Treatment

**Figure 3:**
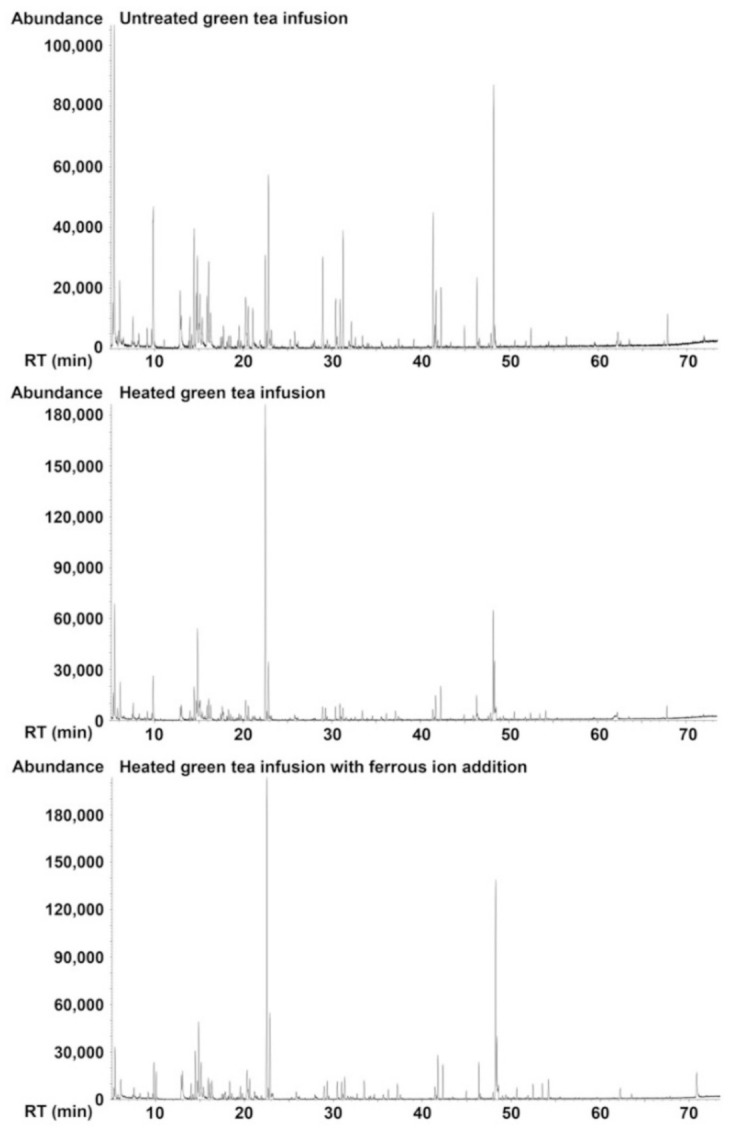
GC-MS total ion count chromatograms of green tea infusion with different treatments.

GC-MS analysis (Figure 3 and Table 5) revealed that 49, 54, and 61 volatiles were detected in unheated tea infusion, heated tea infusion, and heated tea infusion with Fe^2+^ addition, respectively. Among them, 42 volatiles were observed in all infusions. It indicated that the majority of volatile components in the three infusions were similar and the presence of Fe^2+^ promoted the formation of new volatiles.

**Figure 4:**
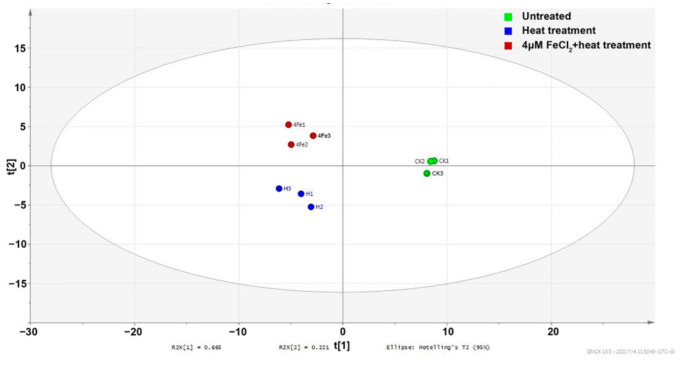
PCA score plot based on volatiles in tea infusions with different treatments.

The volatile profiles of the three infusions were quite different. It was supported by the results of PCA. Points representing samples with different treatments in the PCA score plot were clearly separated (Figure 4). The types and relative abundance of alkenes, alcohols, and heterocycles were gradually increased in unheated tea infusion, heated tea infusion, and heated tea infusion with Fe^2+^ addition, respectively. Meanwhile the relative abundance of aldehydes, ketones, and esters were gradually decreased in the three infusions, respectively. When categorized by aroma characteristics, green/grassy volatiles were gradually decreased and woody volatiles were gradually increased in the three infusions, respectively.

A total of 28 volatiles were decreased after heat treatment. Eleven of them were further decreased in heated tea infusion with Fe^2+^ addition, most of which had green/grassy scents. Twenty-one volatiles were increased after heat treatment. Five of them were further increased in heated tea infusion with Fe^2+^ addition. They smelt sweet, fruity, or woody. Notably, there were nine volatiles only detected in the heated infusion with Fe^2+^ addition and their aroma characteristics were so different from each other, ranging from fruity, floral, minty, fresh, herbal, to woody. The loss of green/grassy volatiles might be associated with the less fresh aroma of green tea infusion. The gain of volatiles with too many different aroma characteristics might lead to an unpleasant complex scent. They together presented a dull scent in heated tea infusion with Fe^2+^ addition.

## 3. Materials and Methods

### 3.1. Reagents

Green tea was purchased from Jiangsu Xinpin Tea Co., Ltd (Changzhou, China). (−)-Epigallocatechin gallate (EGCG), (−)-gallocatechin gallate (GCG), (−)-epicatechin (EC), (+)-catechin (C), (−)-epicatechin gallate (ECG), (−)-catechin gallate (CG), (−)-epigallocatechin (EGC), (−)-gallocatechin (GC), and gallic acid (GA) were obtained from Sigma-Aldrich Co. LLC (Saint Louis, MO, USA). Polyvinylpolypyrrolidone (PVPP) and ferrous chloride (FeCl_2_) were purchased from Shanghai Aladdin Bio-Chem Technology Co., Ltd (Shanghai, China). The 30% H_2_O_2_ was purchased from Sinopharm group Co., Ltd (Beijing, China).

### 3.2. Preparation of Green Tea Infusion

Green tea was brewed with deionized water (1:100, *w*/*v*) at 60 °C for 20 min and filtered through a 0.45 μm membrane to obtain tea infusion.

To assess the effects of Fe^2+^ on the aroma quality, degradation of catechins, and generation of H_2_O_2_ of green tea infusion with heat treatment, FeCl_2_ solution was added to green tea infusion to reach the final concentrations of 0, 2, and 4 μmol/L (μM). According to the requirement of the standards for drinking water quality (GB 5749-2006), the ferrous ion concentration in drinking water shall not exceed 5.36 μM. Therefore, the Fe^2+^ concentrations used for experiments in the study were defined as 2 and 4 μM.

**Figure 5:**
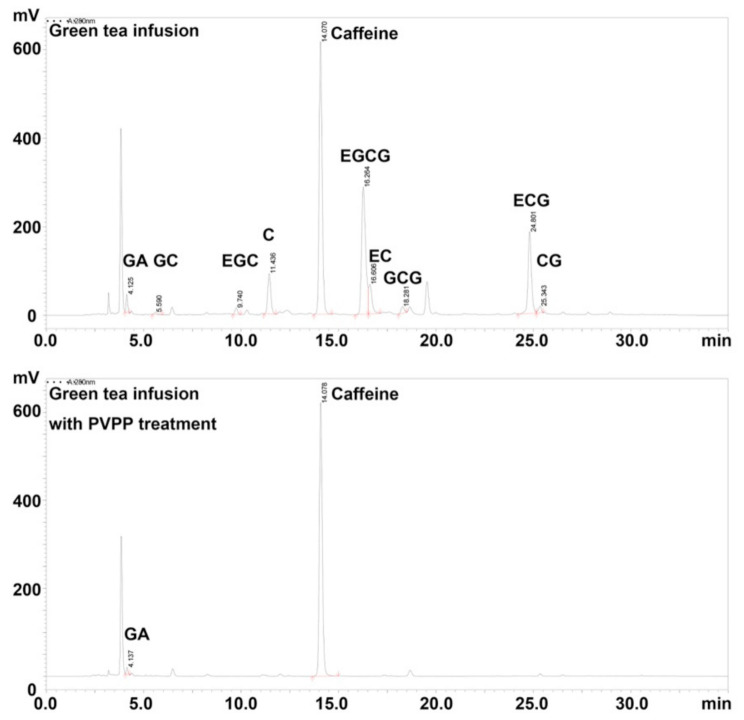
HPLC spectrums of catechins in green tea infusion with and without olyvinylpolypyrrolidone (PVPP) treatment.

To investigate the effect of catechins on Fe^2+^-mediated aroma deterioration, green tea infusion was mixed with PVPP (5%, *w*/*w*) to remove catechins. After PVPP treatment, catechins in green tea infusion were no longer detectable using the HPLC method [13] (Figure 5). FeCl_2_ solution was added to the catechins-removed green tea infusion to reach the final concentrations of 0, 2, and 4 μM.

To investigate the effect of H_2_O_2_ on Fe^2+^-mediated aroma deterioration, H_2_O_2_ was added to the catechins-removed green tea infusion to reach the final concentration of 100 μM.

Infusions with different additions were incubated at 100 °C for 40 min and cooled to room temperature for further tests.

### 3.3. Sensory Evaluation of Green Tea Infusion

The aroma quality of tea infusion was assessed according to the national standard GB/T 21733-2008, and scored by a team of seven qualified panelists (three men and four women, 25–50 years old), all of whom achieved certificates for tea-quality evaluation from the Tea Scientific Society of China. A ten-point scale was used for scoring. The score 0 is for least acceptable and 10 is for most acceptable [14].

### 3.4. Determination of Catechins and GA in Green Tea Infusion

The concentrations of catechins and GA were measured by an established HPLC method [13].

The separation was performed on a Symmetry C_18_ column (5 μm, 4.6 mm × 250 mm, Waters, Milford, MA, USA) using a Shimadzu LC-20A system (Shimadzu, Tokyo, Japan) equipped with a UV detector set at 280 nm. The gradient separation was carried out using 2% acetic acid in water and acetonitrile as mobile phases A and B, with the flow rate at 1 mL/min for 30 min and the column temperature at 35 °C. Separation was conducted under the following conditions: 0–16 min, 6.5–15% B; 16–25 min, 15–25% B; and 25–30 min, and 25–6.5% B.

GA (3.54–56.7 mg/L), EGCG (3.23–51.7 mg/L), GCG (5.00–80.0 mg/L), EC (5.31–85.0 mg/L), C (5.31–85.0 mg/L), ECG (4.11–65.8 mg/L), CG (1.56–25.0 mg/L), EGC (5.21–83.3 mg/L), and GC (2.55–40.8 mg/L) were used as standards to prepare calibration curves.

### 3.5. Determination of H_2_O_2_ in Green Tea Infusion

The levels of H_2_O_2_ were determined using a Hydrogen Peroxide Assay Kit (Beyotime Biotechnology, Nantong, China) according to the manufacturer’s instructions. Briefly, 50 μL of the infusion was added to a 96-well plate, mixed with 100 μL of the hydrogen peroxide reaction reagent, stayed at room temperature for 30 min, and then the absorbance was read at 560 nm using the BioTek Synergy 2 multi-mode microplate reader (Winooski, VT, USA).

### 3.6. Analysis of Volatiles in Green Tea Infusion

Volatiles in tea infusion were determined by gas chromatography–mass spectrometry (GC-MS) as previously reported [15].

Thirty milliliters of the infusion containing ethyl caprate as the internal standard was added into a 50 mL glass vial. The glass vial was sealed and incubated at 60 °C. A SPME needle (Supelco, Bellefonte, PA, USA) was used to absorb volatiles for 60 min. Then the SPME needle was inserted into the injection port of GC and volatiles were desorbed at 250 °C for 5 min.

Volatiles were analyzed using an Agilent6890 gas chromatograph coupled with an Agilent HP 5973 mass selective detector (Agilent, Wilmington, DE, USA). The separation was performed on a DB-5MS capillary column (30 m × 250 μm × 0.25 μm). The GC condition was as follows: the GC inlet temperature of 250 °C, and the split ratio of 15:1, the carrier gas (high purity helium) flow of 1 mL/min. The separation was conducted under the following conditions: 0–2 min, 40 °C; 2–24.5 min, 40–85 °C; 24.5–26.5 min, 85 °C; 26.5–64.5 min, 85–180 °C; 64.5–66.5 min, 180 °C; 66.5–71.5 min, 180–230 °C; and 71.5–73.5 min, 230 °C.

For MS analysis, the temperature of the ion source was 230 °C, the voltage was 70 eV and the scan range was 40 to 400 m/z.

Tentative identification of volatiles was made by comparing the MS fragmentation patterns with data from the National Institute for Standards and Technology database (NIST 08, match percentage >80%). The relative abundance of each compound was calculated by comparing the peak area of each compound to the total peak area.

### 3.7. Statistical Analysis

Data are presented as the mean ± standard error of the mean (SEM). All experiments were carried out in triplicate. The principal component analysis (PCA) was performed using SIMCA-P 13.0 software (Umetric, Umea, Sweden). The results were analyzed with SPSS Version 18.0 for Windows using the one-way analysis of variance with 2-sided Dunnett’s post hoc test to determine overall differences between groups. *p*-values < 0.05 were considered to be statistically significant.

## 4. Conclusions

**Figure 6:**
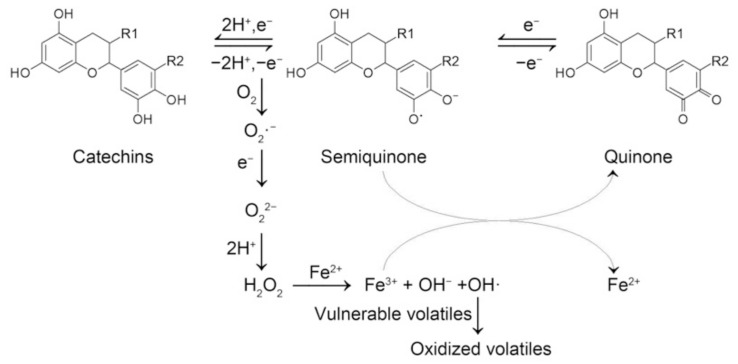
Proposed reaction scheme for the effect of ferrous ion on heat-induced aroma deterioration of green tea infusion.

Fe^2+^ significantly exacerbated the aroma quality of green tea infusion with heat treatment. It promoted the degradation of catechins and the generation of H_2_O_2_. Catechins were prerequisites for Fe^2+^-mediated aroma deterioration. They were important sources of H_2_O_2_. H_2_O_2_ alone showed limited effects on aroma. Fe^2+^ and H_2_O_2_ worked together to induce aroma deterioration. Fe^2+^ significantly modified the volatile profiles of green tea infusion. Green/grassy volatiles were reduced and several volatiles with different aroma characteristics were increased or newly generated, leading to a dull scent. Based on the above results and previous references [12,16,17], a reaction scheme was proposed (Figure 6). It was deduced that under heat treatment, catechins could deprotonate and lose electrons to form semiquinone and quinone, which was reversible. The electrons reacted with dissolved oxygen and hydrogen ions, leading to the generation of H_2_O_2_. The presence of Fe^2+^ triggered the Fenton reaction and produced multiple free radicals, including hydroxyl radicals, which were regarded as the most active radicals and were capable of oxidizing vulnerable volatiles. Fe^3+^ produced during the process could be rapidly reduced to Fe^2+^ by EGCG, which promoted the transformation of semiquinone to quinone. Taken together, the presence of Fe^2+^ might promote the degradation of catechins to generate H_2_O_2_ and reacted with H_2_O_2_ to produce highly active radicals to deteriorate the aroma of green tea infusion. Our results partially revealed how Fe^2+^ induced aroma deterioration of green tea infusion with heat treatment and implied that elimination of Fe^2+^ in green tea infusion during processing might help to improve the sensory quality of ready-to-drink tea.

## Figures and Tables

**Table 1:** Effect of Fe^2+^ on aroma quality of green tea infusion with heat treatment.

Treatment	Description	Score
Untreated	Fresh	8.7 ± 0.2 ^a^
Heat treatment	Slightly dull	7.0 ± 0.4 ^b^
2 μM FeCl_2_ + Heat treatment	Slightly dull	6.5 ± 0.5 ^b^
4 μM FeCl_2_ + Heat treatment	Dull	5.5 ± 0.4 ^c^

The description and score of infusions were given by a team of qualified panelists. A higher score indicates a better aroma acceptability. The same letter within each column indicates no significant difference (*p >* 0.05).

**Table 2:** Effect of Fe^2+^ on the transformation of catechins in green tea infusion with heat treatment.

Concentrations (μg/mL)	Untreated	Heat Treatment	2 μM FeCl_2_ + Heat Treatment	4 μM FeCl_2_ + Heat Treatment
GA	4.41 ± 0.19 ^a^	12.6 ± 0.8 ^b^	13.0 ± 0.8 ^b^	13.4 ± 1.1 ^b^
GC	18.6 ± 0.1 ^a^	41.6 ± 1.3 ^b^	38.3 ± 0.6 ^c^	37.3 ± 0.1 ^d^
EGC	53.9 ± 3.6 ^a^	52.6 ± 2.7 ^a^	50.6 ± 3.3 ^a^	49.5 ± 2.0 ^a^
C	5.14 ± 0.18 ^a^	12.0 ± 0.8 ^b^	11.2 ± 0.1 ^b^	11.5 ± 0.2 ^b^
EGCG	818 ± 9 ^a^	582 ± 5 ^b^	568 ± 22 ^bc^	561 ± 5 ^c^
EC	45.5 ± 1.8 ^a^	33.2 ± 0.5 ^b^	33.0 ± 1.5 ^b^	32.9 ± 0.6 ^b^
GCG	5.07 ± 0.25 ^a^	219 ± 4 ^b^	200 ± 10 ^c^	197 ± 10 ^c^
ECG	93.3 ± 0.2 ^a^	95.7 ± 2.1 ^a^	93.7 ± 1.0 ^a^	92.9 ± 3.0 ^a^
CG	6.10 ± 0.11 ^a^	16.9 ± 0.2 ^b^	15.6 ± 1.0 ^bc^	15.5 ± 0.9 ^c^
Total catechins	(1.05 ± 0.02) ×10^3 a^	(1.05 ± 0.01) ×10^3 a^	(1.01 ± 0.02) × 10^3 b^	998 ± 6 ^b^
Non-gallated catechins	123 ± 5 ^a^	139 ± 5 ^b^	133 ± 6 ^ab^	131 ± 3 ^a^
Gallated catechins	923 ± 9 ^a^	914 ± 7 ^a^	877 ± 10 ^b^	867 ± 9 ^b^
Non-epi-catechins	35.0 ± 0.2 ^a^	290 ± 6 ^b^	265 ± 11 ^c^	262 ± 11 ^c^
Epi-catechins	(1.01 ± 0.01) ×10^3 a^	763 ± 6 ^b^	745 ± 26 ^bc^	737 ± 5 ^c^

Non-gallated catechins referred to GC, EGC, C, and EC, while gallated catechins referred to GCG, EGCG, CG, and ECG. Non-epi-catechins referred to GC, C, GCG, and CG, while epi-catechins referred to EGC, EC, EGCG, and ECG. The experiments were carried out in triplicate. The same letter within each row indicates no significant difference (*p* > 0.05).

**Table 3:** Effect of Fe^2+^ on aroma quality of catechins-removed green tea infusion.

Treatment	Score
Untreated	8.2 ± 0.3 ^a^
Heat treatment	7.6 ± 0.4 ^ab^
2 μM FeCl_2_ + Heat treatment	7.6 ± 0.4 ^ab^
4 μM FeCl_2_ + Heat treatment	7.3 ± 0.5 ^b^

The same letter within each column indicates no significant difference (*p* > 0.05).

**Table 4:** Effect of H_2_O_2_ on Fe^2+^-mediated aroma deterioration in catechins-removed green tea infusion.

Treatment	Score
Untreated	8.2 ± 0.3 ^a^
Heat treatment	7.5 ± 0.6 ^a^
100 μM H_2_O_2_ +Heat treatment	7.5 ± 0.5 ^a^
4 μM FeCl_2_ + Heat treatment	7.3 ± 0.6 ^a^
4 μM FeCl_2_ + 100 μM H_2_O_2_ +Heat treatment	6.0 ± 0.4 ^b^

The same letter within each column indicates no significant difference (*p >* 0.05).

**Table 5:** Effect of Fe^2+^ on the volatile profile of green tea infusion with heat treatment.

RT	CAS No.	Molecular Formula	Name	Relative Abundance (%)			Aroma Properties
				Unheated	Heat Treatment	4 μM FeCl_2_ + Heat Treatment	
5.310	141-79-7	C_6_H_10_O	Mesityl oxide	1.27 ± 0.05 ^a^	0.68 ± 0.05 ^b^	<LOQ ^c^	Pungent
5.437	66-25-1	C_6_H_12_O	Hexanal	11.70 ± 0.92 ^a^	3.86 ± 0.53 ^b^	3.39 ± 0.13 ^b^	Green, grassy
5.764	617-92-5	C_6_H_9_N	1-Ethylpyrrole	0.42 ± 0.05 ^a^	0.32 ± 0.01 ^b^	0.19 ± 0.05 ^c^	
5.944	123-86-4	C_6_H_12_O_2_	Butyl acetate	0.49 ± 0.06 ^a^	<LOQ ^b^	<LOQ ^b^	Fruity banana
7.557	544-12-7	C_6_H_12_O	Trans-3-hexen-1-ol	1.06 ± 0.03 ^a^	0.71 ± 0.07 ^b^	0.58 ± 0.03 ^c^	Green
9.654	6728-31-0	C_7_H_12_O	(Z)-4-Heptenal	0.48 ± 0.03 ^a^	0.29 ± 0.05 ^b^	0.19 ± 0.05 ^c^	Fatty, green, creamy
9.818	111-71-7	C_7_H_14_O	Heptanal	3.67 ± 0.28 ^a^	1.98 ± 0.41 ^b^	2.00 ± 0.24 ^b^	Fruity
9.843	222866	C_8_H_9_NO_2_	Oxime-, methoxy-phenyl-	1.56 ± 0.07 ^a^	0.99 ± 0.19 ^b^	1.31 ± 0.18 ^ab^	
12.882	57266-86-1	C_7_H_12_O	(Z)-2-Heptenal	2.17 ± 0.13 ^a^	1.41 ± 0.05 ^b^	1.49 ± 0.10 ^b^	Oily
12.998	100-52-7	C_7_H_6_O	Benzaldehyde	1.44 ± 0.08 ^a^	1.36 ± 0.02 ^a^	2.34 ± 0.22 ^b^	Strong sharp almond aroma
13.988	26456-76-8	C_9_H_18_	3,5,5-Trimethyl-2-hexene	1.10 ± 0.10 ^a^	0.81 ± 0.02 ^b^	0.83 ± 0.03 ^b^	
14.183	2918-13-0	C_7_H_12_O	1-Hepten-3-one	0.44 ± 0.06 ^a^	0.28 ± 0.04 ^b^	0.33 ± 0.00 ^c^	Metallic
14.442	3391-86-4	C_8_H_16_O	1-Octen-3-ol	4.55 ± 0.05 ^a^	2.86 ± 0.23 ^b^	2.72 ± 0.16 ^b^	Mushroom aroma, earthy
14.693	110-93-0	C_8_H_14_O	5-Hepten-2-one, 6-methyl-	2.19 ± 0.14 ^a^	1.38 ± 0.08 ^b^	1.13 ± 0.08 ^c^	Citrus and lemongrass aroma
14.822	3050-69-9	C_8_H_14_O_2_	Vinyl hexanoate	4.14 ± 0.65 ^a^	5.65 ± 0.47 ^b^	4.79 ± 0.27 ^a^	Fruity, sweet
15.366	13643-08-8	C_8_H_14_	2,4-Octadiene	1.05 ± 0.30 ^ab^	0.72 ± 0.07 ^a^	0.91 ± 0.11 ^b^	
15.903	124-13-0	C_8_H_16_O	Octanal	2.15 ± 0.01 ^a^	1.45 ± 0.08 ^b^	1.36 ± 0.00 ^c^	Orange peel, fatty
16.082	3681-71-8	C_8_H_14_O_2_	cis-3-Hexenyl acetate	3.58 ± 0.15 ^a^	1.10 ± 0.12 ^b^	1.02 ± 0.06 ^b^	Green, unripe banana
16.297	4313-03-5	C_7_H_10_O	(E,E)-2,4-Heptadienal	1.81 ± 0.02 ^a^	1.37 ± 0.05 ^b^	1.41 ± 0.11 ^b^	Fatty, green, oily
17.473	5989-27-5	C_10_H_16_	D-Limonene	<LOQ ^a^	<LOQ ^a^	0.43 ± 0.09 ^b^	Citrus, orange
17.633	411448	C_8_H_18_O	2-Ethyl-1-hexanol	0.51 ± 0.15 ^a^	<LOQ ^b^	0.22 ± 0.07 ^c^	Citrus aroma
17.764	100-51-6	C_7_H_8_O	Benzyl alcohol	<LOQ ^a^	<LOQ ^a^	0.58 ± 0.22 ^b^	Floral, rose
17.768	2408-37-9	C_9_H_16_O	2,6,6-Trimethylcyclohexanone	0.92 ± 0.01 ^a^	<LOQ ^b^	<LOQ ^b^	Pungent
18.322	122-78-1	C_8_H_8_O	Benzene acetaldehyde	0.43 ± 0.02 ^a^	0.97 ± 0.06 ^b^	1.15 ± 0.13 ^b^	Sweet floral, hyacinth aroma
18.551	2167-14-8	C_7_H_9_NO	1H-Pyrrole-2-carboxaldehyde, 1-ethyl-	0.44 ± 0.04 ^a^	0.33 ± 0.06 ^b^	0.37 ± 0.05 ^ab^	Burnt, roasted, smoky
19.223	65405-80-3	C_10_H_16_O_2_	(E,Z)-2-Butenoic acid, 3-hexenyl ester	<LOQ ^a^	<LOQ ^a^	0.13 ± 0.01 ^b^	Green vegetable
19.377	78-59-1	C_9_H_14_O	Isophorone	0.27 ± 0.01 ^a^	<LOQ ^b^	0.11 ± 0.00 ^c^	Cooling woody
19.515	2548-87-0	C_8_H_14_O	(E)-2-Octenal	0.79 ± 0.06 ^a^	0.68 ± 0.05 ^a^	0.74 ± 0.07 ^a^	Fresh cucumber aroma
19.731	98-86-2	C_8_H_8_O	Acetophenone	0.35 ± 0.02 ^a^	0.44 ± 0.06 ^b^	0.46 ± 0.01 ^b^	Sweet, almond
20.275	38284-27-4	C_8_H_12_O	3,5-Octadien-2-one	2.08 ± 0.34 ^a^	1.91 ± 0.64 ^ab^	1.59 ± 0.01 ^b^	Fruity, fatty, mushroom aroma
20.556	111-87-5	C_8_H_18_O	1-Octanol	1.72 ± 0.02 ^a^	1.22 ± 0.03 ^b^	1.38 ± 0.22 ^b^	Waxy, orange
21.889	30086-02-3	C_8_H_12_O	(E,E)-3,5-Octadien-2-one	0.25 ± 0.06 ^a^	0.23 ± 0.03 ^a^	0.25 ± 0.03 ^a^	Green, grassy
22.477	78-70-6	C_10_H_18_O	Linalool	3.45 ± 0.16 ^a^	19.79 ± 0.68 ^b^	19.99 ± 0.89 ^b^	Floral
22.673	29957-43-5	C_10_H_16_O	3,7-Dimethylocta-1,5,7-trien-3-ol	0.68 ± 0.11 ^a^	0.64 ± 0.04 ^a^	0.63 ± 0.05 ^a^	Mouldy
22.836	124-19-6	C_9_H_18_O	Nonanal	6.57 ± 0.93 ^ab^	6.84 ± 0.18 ^a^	5.62 ± 0.24 ^b^	Waxy, rosy, orange peel aroma
22.991	60-12-8	C_8_H1_0_O	Phenylethyl Alcohol	0.35 ± 0.00 ^a^	0.52 ± 0.04 ^b^	0.48 ± 0.01 ^b^	Floral, rose
28.922	119-36-8	C_8_H_8_O_3_	Methyl salicylate	<LOQ ^a^	<LOQ ^a^	0.51 ± 0.13 ^b^	Wintergreen mint
28.960	53398-84-8	C_10_H_18_O_2_	Butanoic acid, 3-hexenyl ester, (E)-	4.33 ± 0.06 ^a^	1.22 ± 0.10 ^b^	0.76 ± 0.10 ^c^	
29.321	98-55-5	C_10_H_18_O	α-Terpineol	<LOQ ^a^	1.12 ± 0.06 ^b^	1.28 ± 0.00 ^c^	Pine, lilac
29.49	116-26-7	C_10_H_14_O	Safranal	<LOQ ^a^	<LOQ ^a^	0.23 ± 0.00 ^b^	Fresh, herbal
30.423	112-31-2	C_10_H_20_O	Decanal	1.47 ± 0.59 ^a^	2.95 ± 0.25 ^b^	1.21 ± 0.07 ^a^	Citrus, sweet floral, waxy
30.903	432-25-7	C_10_H_16_O	β-Cyclocitral	1.91 ± 0.01 ^a^	1.19 ± 0.08 ^b^	1.13 ± 0.00 ^b^	Woody
31.059	496-16-2	C_8_H_8_O	2,3-Dihydrobenzofuran	<LOQ ^a^	0.24 ± 0.01 ^b^	0.34 ± 0.03 ^c^	Sweet, nut
31.253	2177-77-7	C_7_H_14_O_2_	Methyl 2-, Methylpentanoate	4.15 ± 0.66 ^a^	0.70 ± 0.14 ^b^	1.37 ± 0.02 ^c^	Fruity
32.183	35852-46-1	C_11_H_20_O_2_	n-Valeric acid cis-3-hexenyl ester	1.06 ± 0.05 ^a^	0.20 ± 0.03 ^b^	<LOQ ^c^	Green fruity, unripe banana
33.450	624-15-7	C_10_H_18_O	3,7-Dimethyl-2,6-octadienol	<LOQ ^a^	0.80 ± 0.11 ^b^	1.09 ± 0.06 ^c^	
35.575	120-72-9	C_8_H_7_N	Indole	0.28 ± 0.01 ^a^	0.34 ± 0.10 ^ab^	0.38 ± 0.06 ^b^	Floral, fecal
36.176	36431-72-8	C_13_H_22_O	Theaspirane	<LOQ ^a^	0.84 ± 0.06 ^b^	0.58 ± 0.00 ^c^	Woody, spicy
37.193		C_13_H_23_O	2,6,10,10-Tetramethyl-1-oxaspiro[4.5]dec-7-ene	<LOQ ^a^	1.27 ± 0.02 ^b^	0.96 ± 0.05 ^c^	
41.403	31501-11-8	C_12_H_22_O_2_	Hexanoic acid, 3-hexenyl ester, (Z)-	5.08 ± 0.19 ^a^	1.27 ± 0.14 ^b^	0.79 ± 0.04 ^c^	Fruity green, pear
41.606	61444-38-0	C_12_H2_0_O_2_	cis-3-Hexenyl cis-3-hexenoate	0.87 ± 0.05 ^a^	0.27 ± 0.00 ^b^	0.19 ± 0.02 ^c^	Green
41.721	488-10-8	C_11_H_16_O	Jasmone	2.09 ± 0.00 ^a^	2.47 ± 0.19 ^b^	2.64 ± 0.01 ^c^	Floral, Jasmin
44.928	3879-26-3	C_13_H_22_O	Nerylacetone	0.58 ± 0.05 ^a^	0.94 ± 0.12 ^b^	0.47 ± 0.08 ^a^	Woody
46.344	79-77-6	C_13_H_20_O	β-Ionone	2.24 ± 0.02 ^a^	1.97 ± 0.16 ^b^	2.14 ± 0.14 ^ab^	Floral, woody
47.931	166273-38-7	C_19_H_30_O_3_	2,4-Ditert-butylphenyl 5-hydroxypentanoate	0.45 ± 0.05 ^a^	0.38 ± 0.07 ^a^	0.41 ± 0.05 ^a^	
48.219	500-66-3	C_11_H_16_O_2_	1,3-Benzenediol, 5-pentyl-	9.56 ± 0.04 ^a^	13.13 ± 1.13 ^b^	13.70 ± 0.21 ^c^	Olive
48.354	483-76-1	C_15_H_24_	(+)-δ-Cadinene	0.79 ± 0.07 ^a^	3.00 ± 0.44 ^b^	4.01 ± 0.29 ^c^	Herbal, woody
48.469	483-77-2	C_15_H_22_	(−)-Calamenene	<LOQ ^a^	<LOQ ^a^	0.73 ± 0.11 ^b^	Herb spice
48.545	41702-63-0	C_15_H_24_	Epizonarene	<LOQ ^a^	<LOQ ^a^	0.69 ± 0.11 ^b^	
49.384	21391-99-1	C_15_H_20_	α-Calacorene	<LOQ ^a^	<LOQ ^a^	0.32 ± 0.10 ^b^	Woody
50.619	142-50-7	C_15_H_26_O	Nerolidol	<LOQ ^a^	0.92 ± 0.28 ^b^	0.51 ± 0.01 ^c^	Floral
52.427	77-53-2	C_15_H_26_O	Cedrol	0.52 ± 0.05 ^a^	0.81 ± 0.02 ^b^	0.88 ± 0.05 ^b^	Cedarwood, woody, soft sweet
53.501	16728-99-7	C_15_H_24_	Naphthalene, 1,2,3,4,4a,7-hexahydro-1,6-dimethyl-4-(1-methylethyl)-	<LOQ ^a^	0.89 ± 0.20 ^b^	0.92 ± 0.06 ^b^	
54.168	19912-67-5	C_15_H_26_O	Epicubebol	<LOQ ^a^	1.23 ± 0.08 ^b^	1.34 ± 0.16 ^b^	
62.562	66408-55-7	C_17_H_28_O	5,9,13-Trimethyl-tetradeca-4,8,12-trienal	<LOQ ^a^	0.33 ± 0.11 ^b^	<LOQ ^a^	
63.552	84-74-2	C_16_H_22_O_4_	Dibutyl phthalate	<LOQ ^a^	<LOQ ^a^	0.30 ± 0.05 ^b^	Faint odor
67.845	57-10-3	C_16_H_32_O_2_	n-Hexadecanoic acid	0.53 ± 0.13 ^a^	<LOQ ^b^	<LOQ ^b^	Waxy, fatty
71.894	83834-59-7	C_18_H_26_O_3_	4-Methoxycinnamic acid 2-ethylhexyl ester	<LOQ ^a^	0.71 ± 0.11 ^b^	<LOQ ^a^	

LOQ is short for the limit of quantification. The experiments were carried out in triplicate. The same letter within each row indicates no significant difference (*p* > 0.05).

## Data Availability

Not applicable.
